# The Relative Safety of Ether and Chloroform

**Published:** 1893-02

**Authors:** W. Roger Williams

**Affiliations:** Late Surgeon to Middlesex Hospital, England


### THE RELATIVE SAFETY OF ETHER AND CHLOROFORM.*

BY W. ROGER WILLIAMS, F. R. C. S.,
Late Surgeon to Middlesex Hospital, England.

One of the most characteristic features of modern science is the demand for greater accuracy.

The vague statements that satisfied our forefathers no longer suffice for us.

When, for instance, it is alleged, that at the Edinburgh Infirmary, during a period of ten years, only one death under chloroform took place in 36,500 administrations, and that during the Crimean War it was administered more than 25,000 times without a single death, instead of accepting these state-

*Selected from the *Medical Chronicle*, December, 1892.

ments on the authority of their authors as our forefathers did, we ask for a detailed account of the data on which they are based.

Then we discover that there are no such data, and that these statements are mere guesses, having no other foundation than the *ipse dixit* of their authors, who ought never to have made them.

It may of course be urged in defence of such extravagant utterances, that, in the absence of reliable information, they were better than nothing at all; for it certainly is a most surprising and not very creditable fact, that with half a century's experience of anæsthetics at our back, there existed until quite lately no really reliable information as to the relative frequency of such accidents.

Anæsthetists seem to have forgotten how to count, not only in our own country, but all over the world. The honorable exception to this dereliction of duty is St. Bartholomew's Hospital, where, for many years past, there has been kept a most admirable record of the administration of anæsthetics and of the fatalities that have occurred.

This is consequently the only really reliable source of information now available for estimating the relative safety of anæsthetics.

Nearly two years ago, thinking the subject well worth a little trouble, I tabulated these records for several years, and a brief statement of the results was published in the *Lancet.*

Since then the subject has continued to attract, in an increasing degree, the attention of the profession.

The object of the present communication is to set forth the results of a more extensive and detailed analysis of the facts up to date; so that, so far as the occurrence of these fatalities is concerned, there shall in the future be no ambiguity.

To this end I have tabulated the hospital records for the 16 years, 1875-90, and the results are set forth in the subjoined tables:

CHLOROFORM.

| Year. | Administrations. | Deaths. |
|---|---|---|
| 1875 | 617 | Nil |
| 1876 | 670 | Nil |
| 1877 | 699 | Nil |
| 1878 | 794 | 1 |
| 1879 | 975 | 1 |
| 1880 | 1,055 | Nil |
| 1881 | 1,072 | 1 |
| 1882 | 1,349 | 2 |
| 1883 | 1,421 | 2 |
| 1884 | 1,244 | Nil |
| 1885 | 1,331 | Nil |
| 1886 | 1,425 | 1 |
| 1887 | 1,702 | 1 |
| 1888 | 1,711 | 1 |
| 1889 | 1,601 | 2 |
| 1890 | 1,860 | 1 |
| Total | 19,526 | 13 |

ETHER.

| Year. | Ether alone. | Deaths. | Ether with Gas. | Deaths. | Total. | Deaths. |
|---|---|---|---|---|---|---|
| 1875 | 120 | Nil | 764 | Nil | 884 | Nil |
| 1876 | 28 | Nil | 1,004 | Nil | 1,032 | Nil |
| 1877 | 23 | Nil | 1,123 | Nil | 1,346 | Nil |
| 1878 | 15 | 1 | 1,009 | Nil | 1,024 | 1 |
| 1879 | 23 | Nil | 984 | Nil | 1,007 | Nil |
| 1880 | 43 | 1 | 1,304 | Nil | 1,347 | 1 |
| 1881 | 85 | Nil | 1,209 | Nil | 1,294 | Nil |
| 1882 | 337 | Nil | 1,076 | 1 | 1,413 | 1 |
| 1883 | 566 | Nil | 1,156 | Nil | 1,722 | Nil |
| 1884 | 1,016 | Nil | 704 | Nil | 1,720 | Nil |
| 1885 | 1,118 | Nil | 386 | Nil | 1,504 | Nil |
| 1886 | 1,109 | Nil | 567 | Nil | 1,676 | Nil |
| 1887 | 1,197 | Nil | 662 | Nil | 1,859 | Nil |
| 1888 | 1,003 | Nil | 349 | Nil | 1,052 | Nil |
| 1889 | 810 | Nil | 509 | Nil | 1,319 | Nil |
| 1890 | 998 | 1 | 135 | Nil | 1,133 | 1 |
| Total | 8,391 | 3 | 12,941 | 1 | 21,332 | 4 |

From these it will be gathered that, during this period, chloroform was administered 19,526 times with 13 deaths, or 1 in 1,502; whereas, during the same period, ether was administered

21,332 times, with only 4 deaths, 1 in 5,333. In 12,941 of the latter cases ether was preceded by "gas," and only one fatality belongs to this category; in the other 8,391 cases ether alone was given and 3 deaths occurred, or 1 in 2,797.

It follows from the foregoing that ether is a very much safer anæsthetic than chloroform; it is, in fact, more than 3½ times as safe; but much safer than either of these agents alone is ether preceded by gas.

I shall be glad if experimental physiologists can explain this phenomenon, as its elucidation may lead to the discovery of a more perfect means of inducing anæsthesia than any we as yet possess.

Of the 13 deaths under chloroform some details are given in 12, as follows:

(1) A man, aged 52, suffering from cellulitis of the leg, was chloroformed to allow incisions being made. Death ensued from syncope, just as the operation was concluded. The necropsy revealed advanced fatty degeneration of the heart.

(2) A man, aged 26, much weakened by a pyo-nephrosis, was given chloroform for the purpose of having a sinus in the loin examined. Before anæsthesia was complete he was seized with syncope, and died in a few minutes. There was no post mortem.

(3) A surgeon, aged 56, admitted for epithelioma of the tongue, in a very debilitated condition, owing to alcoholism, neglect, and hemorrhage from the diseased part. Chloroform was given for the purpose of removing the tongue. During its administration the patient became exceedingly violent, and bleeding from the tongue recommenced. Before the operation was completed he turned pale, became suddenly livid, and then ceased to breathe. No respiratory movements were afterwards made, although inversion and other restorative methods were employed. At the post mortem the right lung was found to be firmly fixed, throughout its whole extent, to the chest wall by old adhesions; there was nothing abnormal about the heart.

(4) A wheelwright, aged 53, admitted for a wound of the forearm and division of the ulna by a circular saw. Under chloroform the ends of the bone were wired together. A week later the part was much inflamed, and it was deemed necessary to make an incision into it. For this purpose chloroform was again administered. Soon after the commencement of the inhalation the pulse became weak and irregular, and the administration was proceeded with cautiously; but before insensibility was complete the patient became suddenly faint and died. At the necropsy the heart was found hypertrophied and dilated, especially on the right side, and the edges of the mitral valve were slightly thickened. Both lungs and the spleen were thickly studded with miliary tubercles, and a few were also found beneath the endocardium. There was commencing cirrhosis of the liver and kidneys.

(5) A man, aged 52, to whom chloroform was administered for excision of epithelioma of the lip, struggled violently while it was being administered, and when his struggles ceased the pulse was imperceptible, and breathing ceased after a few respirations.

(6) A man, aged 30, died of asphyxia while under the influence of chloroform, given for the setting of a double fracture of the jaw. Artificial respiration and tracheotomy were of no avail. No post mortem.

(7) A boy, aged 11, died of syncope after the administration of chloroform.

(8) A male child, 1½ years old, died under chloroform while a diseased ankle was being put in a splint. At the necropsy the heart was found to be greatly dilated and hypertrophied without apparent cause

(9) A feeble child, eight months old, died of syncope during the administration of chloroform for the performance of an operation for harelip. Respiration continued after the heart had ceased to beat. Post mortem showed general hypertrophy of the heart with dilation of the left ventricle, but no valvular disease or malformation.

(10) A woman, aged 72, died of syncope under chloroform, which was administered for the excision of a glaucomatous eyeball. At the necropsy the heart was found to be large (14 oz.) and fatty.

(11) A woman, aged 50, died under chloroform, which was administered for the opening of an abscess in the thigh, before the operation was commenced. The pulse failed first. She was subject to bronchitis, and her circulation was feeble.

(12) The only particulars given of this case is that the patient died of syncope under chloroform.

The most important fact brought out by these reports is the great frequency of death from syncope, no less than eleven out of twelve deaths being due to this cause. The universal testimony of clinical experience is to the same effect.

It is impossible for clinicians to abandon the evidence of their senses in this matter at the dictum of the Hyderabad Commission.

On post mortem examination, in six of the above fatal cases, the heart was found to be extensively diseased in all but one; in two it was in an advanced state of fatty degeneration, and in three it was markedly hypertrophied and dilated. Hence it may be concluded that morbus cardis adds greatly to the risks of chloroformisation.

Three of those who died of syncope were the subjects of pulmonary disease, and one of these had heart disease as well.

Such fatalities mostly occur before the commencement of the operation, and while anæsthesia is incomplete. This was the case in six out of ten cases. In two cases the accident occurred while the operation was in progress, and in two cases just after its completion.

Men seem to be much more liable to these accidents than women, and no age is exempt.

I will now give a brief account of the three deaths under ether.

(1) A man, aged 56, came under treatment for fractured tibia and fibula. He was a drunkard, and soon became subject to delirium tremens. The day after his admission ether was administered, in order to readjust the displaced broken bones. This was satisfactorily done, when, a few minutes after the cessation of the inhalation, the heart suddenly stopped beating, the respiration ceased, and the patient died. At the necropsy the lungs were found to be much engorged, the heart was flabby, and the liver in fatty degeneration.

(2) A man, aged 61, admitted in a very weak state, suffering from strangulated inguinal hernia and constant vomiting. During the administration of ether the pulse became imperceptible, and respiration ceased. On post mortem examination the heart was slightly fatty, and its cavities were empty. The lungs were emphysematous, and engorged posteriorly.

(3) A man, aged 47, in a state of profound collapse from intestinal obstruction, was given ether in order that lumbar colotomy might be performed. He vomited frequently during the progress of its administration, and after the inhalation had been continued for about ten minutes he became livid and never rallied again. There was no post mortem.

The only fatality under the influence of gas and ether was in the case of a very anæmic woman, aged 21, whose pulse was very weak. She died suddenly, shortly after the commencement of the administration. The necropsy revealed extensive effusion into the right pleura, with displacement of the heart to the left. Both ureters were dilated and suppurating. The liver and uterus were the seats of large hydatid cysts.

Thus in all these deaths under ether the patients were the subjects of exceedingly grave disease, which had dangerously depressed the heart's action.

---

A prescription given by Prof. Bartholow, and very highly recommended by Prof. Wilson as being useful in cases of *Enteric Fever*, and which, it is claimed, reduces the severity of the disease, is the following, to be administered from the onset of the disease and throughout its course :

℞. Tinct. iodinii, gtt. ij.
Acid. carbolic., gtt. j.

M. Sig.—To be given in a wineglassful of water every two or three hours.—*College and Clinical Record.*

